# Cryo-electron tomography: moving towards revealing the viral life cycle of *Rice dwarf virus*

**DOI:** 10.1107/S090904951302219X

**Published:** 2013-10-02

**Authors:** Naoyuki Miyazaki, Fusamichi Akita, Atsushi Nakagawa, Kazuyoshi Murata, Toshihiro Omura, Kenji Iwasaki

**Affiliations:** a National Institute for Physiological Sciences, 38 Nishigonaka, Myodaiji, Okazaki, Aichi 444-8585, Japan; b Institute for Protein Research, 3-2 Yamadaoka, Suita, Osaka 565-0871, Japan; c National Agricultural Research Center, 3-1-1 Kannondai, Tsukuba, Ibaraki 305-8666, Japan; d Division of Bioscience, Graduate School of Natural Science and Technology, Faculty of Science, Okayama University, Okayama 700-8530, Japan

**Keywords:** *Rice dwarf virus*, *Phytoreovirus*, virus structure, cryo-electron tomography, cryo-electron microscopy

## Abstract

It is well known that viruses utilize the host cellular systems for their infection and replication processes. However, the molecular mechanisms underlying these processes are poorly understood for most viruses. To understand these molecular mechanisms, it is essential to observe the viral and virus-related structures and analyse their molecular interactions within a cellular context. Cryo-electron microscopy and tomography offer the potential to observe macromolecular structures and to analyse their molecular interactions within the cell. Here, using cryo-electron microscopy and tomography, the structures of *Rice dwarf virus* are reported within fully hydrated insect vector cells grown on electron microscopy grids towards revealing the viral infection and replication mechanisms.

## Abstract 2

The viral and virus-related structures of *Rice dwarf virus* have been visualized by cryo-electron microscopy and tomography revealing the viral infection and replication mechanisms.

## 1. Introduction

*Rice dwarf virus* (RDV), a member of the family *Reoviridae*, is an icosahedral double-layered particle of approximately 70 nm in diameter (Nakagawa *et al.*, 2003 ▶; Miyazaki *et al.*, 2005 ▶), and the agent of rice dwarf disease causing economic damage in many Asian countries. RDV has a 12-segmented dsRNA genome encoding 12 viral proteins. The RDV particle is composed of seven structural proteins (P1, P2, P3, P5, P7, P8 and P9). The P3 structural proteins form the inner capsid shell which encapsidates the viral genome and the P1, P5 and P7 proteins are required for transcription (Hagiwara *et al.*, 2003 ▶, 2004 ▶; Miyazaki *et al.*, 2010*b* ▶). The inner capsid shell is surrounded by the outer capsid shell, which consists of P2, P8 and P9 proteins (Omura & Yan, 1999 ▶). Five kinds of non-structural proteins (Pns4, Pns6, Pns10, Pns11 and Pns12) are related to viral replication within the host cell, including intra-cellular trafficking (Wei *et al.*, 2006*b* ▶), synthesis of viral genome and proteins, assembly of progeny viruses (Wei *et al.*, 2006*c* ▶; Shimizu *et al.*, 2009 ▶), viral release from infected cells (Wei *et al.*, 2008 ▶, 2009 ▶; Miyazaki *et al.*, 2010*a* ▶.), and inter-cellular transport (Wei *et al.*, 2006*a* ▶). However, the structural details underlying these controlled events are poorly understood for RDV and most other viruses. Cryo-electron microscopy (cryo-EM) and tomography (cryo-ET) can be used to observe hydrated cells in a close-to-native state at molecular resolution, which allows analysis of molecular interactions within a cell (Robinson *et al.*, 2007 ▶). Here, we show one of the workable strategies using cryo-EM/ET to study viral infection and replication mechanisms.

## 2. Materials and methods

### 2.1. Cell culture and virus infection on EM grids, and rapid freezing

NC24 cells, originally established from embryonic fragments dissected from the eggs of the leafhopper *Nephotettix cincticeps* (the insect vector of RDV), were maintained in monolayer culture at 298 K in growth medium that was prepared as previously described (Kimura, 1986 ▶). To examine the virus-infected NC24 cells by cryo-EM/ET, the NC24 cells were grown on Quantifoil holey carbon supported gold EM grids. The cells were further cultivated for three to five days after inoculation with RDV at viral dilutions required to reach 100% infection (Kimura, 1986 ▶; Wei *et al.*, 2006*a* ▶), and were plunged frozen in liquid ethane and embedded in vitreous ice using a Vitrobot (FEI, The Netherland).

### 2.2. Cryo-EM/ET observations

The EM grids were stored in liquid nitrogen and transferred into a cryo-electron microscope (Titan-Krios; FEI, The Netherlands). The Titan-Krios was operated at 200 kV, and incorporated a field emission gun, a liquid-nitrogen stage and a Gatan 4096 × 4096 CCD camera (Model UltraScan 4000; Gatan, USA). The cryo-EM images were recorded under low-dose conditions (∼20 e^−^ Å^−2^). To determine three-dimensional structure by cryo-ET, a tilt-series was collected from −60° to +60° with an angular increment of 2° on the Gatan 4096 × 4096 CCD camera in the Titan-Krios. The total electron dose in the acquisition of the tilt series was kept under 60 e^−^ Å^−2^. The tilt series was aligned using 10 nm colloidal gold particles as fiducial markers. Three-dimensional reconstructions were calculated using the software *IMOD* (Kremer *et al.*, 1996 ▶).

## 3. Results and discussion

### 3.1. Cryo-EM/ET observations of the cellular structures under close-to-native conditions

**Figure 1:**
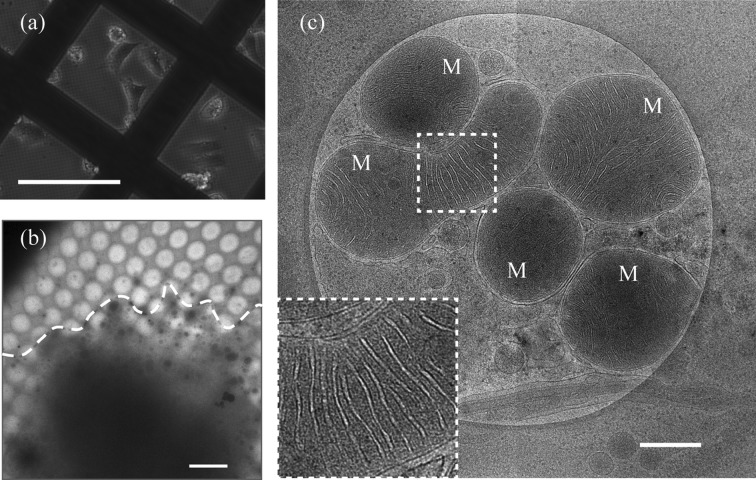
Observations of NC24 cells (the insect vector cells of RDV) cultivated on EM grids. (*a*) A light microscopy image of RDV-infected NC24 cells grown on an EM grid. Bar: 100 µm. (*b*) Low-magnification cryo-EM image of the NC24 cell cultured on the EM grid. The cell is outlined with a white dashed line. The cell periphery was thin enough to allow observation of the cellular structures, while the central part of the cell was too thick for imaging and appeared as a dark region. Bar: 4 µm. (*c*) High-magnification cryo-EM image of the NC24 cells cultivated on the EM grid. The fine membrane structures of mitochondria (M) were clearly visible. (Inset) Enlarged view of the boxed region. Bar: 400 nm.

Electron microscopy (EM) has long been used for high-resolution analysis of cellular and viral structures. Conventional EM sample preparation methods consist of chemical fixation, dehydration and resin embedding of biological specimens. The resin-embedded specimens are then cut into ultra-thin sections, and examined by a transmission electron microscope after staining with electron-dense heavy metals. However, the conventional EM methods cause structural rearrangements, aggregation of cellular contents, and loss of unfixed materials during the sample preparation process, which impedes high-resolution structural analysis. Compared with the conventional method, the advantage of cryo-EM/ET is that the specimens are observed in fully hydrated conditions, which avoids the chemical fixation, dehydration, resin embedding and staining processes. Thus, it limits structural rearrangements or aggregation during the sample preparation as much as possible, which allows us to analyse the fine cellular structures in a close-to-native state. In this study, we used cryo-EM/ET to analyse the structures of the RDV particles within the insect vector (NC24) cells. The NC24 cells adhered to the carbon supported film on the EM grid, and were maintained on the EM grid for the required time for RDV to replicate (Fig. 1*a* ▶). When the cells were examined by cryo-EM, the cellular structures at the periphery were clearly visible (Fig. 1*b* ▶). Dark spherical densities at the edge of the cells, as seen in Fig. 1(*b*) ▶, correspond to the cellular vesicles or organelles. Moreover, the cells were well vitrified without any ice crystal formation in the specimen, and the fine cellular structures were well preserved. Examined at higher magnification, we were able to observe the fine cellular structures in detail. For example, in Fig. 1(*c*) ▶, the fine membrane structures of mitochondria are clearly visualized by cryo-EM.

### 3.2. Cryo-EM/ET observations of the virus structures within the host cells

**Figure 2:**
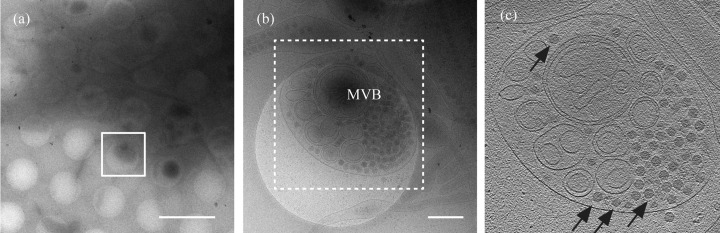
Cryo-EM/ET observations of the RDV structures within the insect vector cell. (*a*) Low-magnification cryo-EM image of the RDV-infected NC24 cell cultured on the EM grid. Bar: 2 µm. (*b*) High-magnification cryo-EM image of the boxed region in (*a*). Bar: 400 nm. (*c*) A slice through the reconstructed tomographic volume obtained from the area highlighted in (*b*). Black arrows indicate the RDV particles within the multivesicular body (MVB).

Viruses must be released from infected cells for successful spreading. In the case of RDV, viral egress from the insect vector cells utilizes multiple pathways without cell lysis. In one of these pathways, multivesicular compartments are involved in the release of RDV particles from the cells (Wei *et al.*, 2008 ▶, 2009 ▶; Miyazaki *et al.*, 2010*a* ▶). The proposed RDV release pathway is that newly synthesized progeny viruses at the viroplasm (Wei *et al.*, 2006*c* ▶) are engulfed by multivesicular compartments that move to the periphery of cells, where they fuse with the plasma membrane to facilitate release of viral particles (Wei *et al.*, 2008 ▶, 2009 ▶). In this study, cryo-EM/ET observations clearly visualized the RDV particles within multi-vesicular bodies at the edge of the cell, which appear to be in a state prior to egress from the infected NC24 cell (Fig. 2 ▶). This result demonstrates the success of our approach using cryo-EM/ET techniques to observe the viral and virus-related structures within cells and to analyse the viral life cycle around the edge of the infected cells. On the other hand, the central part of the cell was very dark, and the cellular and viral structures were invisible, due to the sample thickness being too large for the electron beam to penetrate (Fig. 1*b* ▶). Thus, the limited thickness range of 0.5–1 µm that is accessible with standard accelerating voltage electron microscopes (under 500 kV) restricts observations to only the extreme edge portions of the cell. In order to study the complete viral life cycle, the whole cell body must be examined by cryo-EM/ET. This restriction may be overcome by using a high-voltage electron microscope, because the highly accelerated electrons (over 500 keV) can penetrate thicker specimens. Alternatively, processing a sample into thin vitreous films using CEMOVIS (cryo-electron microscopy of vitreous sections; Al-Amoudi *et al.*, 2005 ▶; Dubochet & Blanc, 2001 ▶) or cryo-FIB (cryo-focused ion beam; Wang *et al.*, 2012 ▶) may allow observation of thicker samples. Cryo-EM/ET observations of such thick specimens are now underway.

## References

[bb1] Al-Amoudi, A., Chang, J. J., Leforestier, A., McDowall, A., Salamin, L. M., Norlen, L. P., Richter, K., Blanc, N. S., Studer, D. & Dubochet, J. (2005). *EMBO J.* **23**, 3583–3588. 10.1038/sj.emboj.7600366 PMC517607 15318169

[bb3] Dubochet, J. & Sartori Blanc, N. (2001). *Micron*, **32**, 91–99. 10.1016/s0968-4328(00)00026-3 10900384

[bb4] Hagiwara, K., Higashi, T., Miyazaki, N., Naitow, H., Cheng, R. H., Nakagawa, A., Mizuno, H., Tsukihara, T. & Omura, T. (2004). *J. Virol.* **78**, 3145–3148. 10.1128/JVI.78.6.3145-3148.2004 PMC353743 14990734

[bb5] Hagiwara, K., Higashi, T., Namba, K., Uehara-Ichiki, T. & Toshihiro, O. (2003). *J. Gen. Virol.* **84**, 1–4. 10.1099/vir.0.18904-0 12655100

[bb6] Kimura, I. (1986). *J. Gen. Virol.* **71**, 1861–1863.

[bb7] Kremer, J. R., Mastronarde, D. N. & McIntosh, J. R. (1996). *J. Struct. Biol.* **116**, 71–76. 10.1006/jsbi.1996.0013 8742726

[bb10] Miyazaki, N., Hagiwara, K., Naitow, H., Higashi, T., Cheng, R. H., Tsukihara, T., Nakagawa, A. & Omura, T. (2005). *J. Mol. Biol.* **345**, 229–237. 10.1016/j.jmb.2004.10.044 15571717

[bb11] Miyazaki, N., Hagiwara, K., Wei, T., Chen, H., Nakagawa, A., Xing, L., Cheng, R. H. & Omura, T. (2010*a*). *J. Gen. Virol.* **91**, 2857–2861. 10.1099/vir.0.022012-0 20631088

[bb12] Miyazaki, N., Wu, B., Hagiwara, K., Wang, C.-Y., Xing, L., Hammar, L., Higashiura, A., Tsukihara, T., Nakagawa, A., Omura, T. & Cheng, R. H. (2010*b*). *J. Biochem.* **147**, 843–850. 10.1093/jb/mvq017 20190042

[bb13] Nakagawa, A., Miyazaki, N., Taka, J., Naitow, H., Ogawa, A., Fujimoto, Z., Mizuno, H., Higashi, T., Watanabe, Y., Omura, T., Cheng, R. H. & Tsukihara, T. (2003). *Structure*, **11**, 1227–1238. 10.1016/j.str.2003.08.012 14527391

[bb14] Omura, T. & Yan, J. (1999). *Adv. Virus Res.* **54**, 15–43. 10.1016/s0065-3527(08)60364-4 10547673

[bb15] Robinson, C. V., Sali, A. & Baumeister, W. (2007). *Nature (London)*, **450**, 973–982. 10.1038/nature06523 18075576

[bb16] Shimizu, T., Yoshii, M., Wei, T., Hirochika, H. & Omura, T. (2009). *Plant Biotechnol. J.* **7**, 24–32. 10.1111/j.1467-7652.2008.00366.x 18761654

[bb17] Wang, K., Strunk, K., Zhao, G., Gray, J. L. & Zhang, P. (2012). *J. Struct. Biol.* **180**, 318–326. 10.1016/j.jsb.2012.07.003 PMC3483467 22796867

[bb18] Wei, T., Hibino, H. & Omura, T. (2008). *J. Gen. Virol.* **89**, 2915–2920. 10.1099/vir.0.2008/002063-0 18931091

[bb19] Wei, T., Hibino, H. & Omura, T. (2009). *Commun. Integr. Biol.* **2**, 324–326. 10.4161/cib.2.4.8335 PMC2734036 19721879

[bb20] Wei, T., Kikuchi, A., Moriyasu, Y., Suzuki, N., Shimizu, T., Hagiwara, K., Chen, H., Takahashi, M., Ichiki-Uehara, T. & Omura, T. (2006*a*). *J. Virol.* **80**, 8593–8602. 10.1128/JVI.00537-06 PMC1563882 16912308

[bb21] Wei, T., Kikuchi, A., Suzuki, N., Shimizu, T., Hagiwara, K., Chen, H. & Omura, T. (2006*b*). *Arch. Virol.* **151**, 1701–1712. 10.1007/s00705-006-0757-4 16609816

[bb22] Wei, T., Shimizu, T., Hagiwara, K., Kikuchi, A., Moriyasu, Y., Suzuki, N., Chen, H. & Omura, T. (2006*c*). *J. Gen. Virol.* **87**, 429–438. 10.1099/vir.0.81425-0 16432031

